# Arginine deiminase: a potential inhibitor of angiogenesis and tumour growth

**DOI:** 10.1038/sj.bjc.6601181

**Published:** 2003-08-26

**Authors:** I-S Park, S-W Kang, Y-J Shin, K-Y Chae, M-O Park, M-Y Kim, D N Wheatley, B-H Min

**Affiliations:** 1 Department of Anatomy, College of Medicine, Inha University, Inchon 400-103, Korea; 2 Department of Pharmacology and BK21 Program for Medical Sciences, College of Medicine, Korea University, Seoul 136-705, Korea; 3 AngioLab, Inc., Taejon 302-735, Korea; 4 Department of Cell Pathology, University of Aberdeen, MacRobert Building, 581 King Street, Aberdeen AB24 5UA, UK

**Keywords:** antiproliferative, antiangiogenic, arginine deiminase, arginine, NO, citrulline

## Abstract

Hydrolysis of plasma arginine to citrulline by arginine deiminase (ADI) was recently shown to suppress lipopolysaccharide-induced nitric oxide (NO) synthesis. Since arginine is the precursor of NO, and the latter modulates angiogenesis, we explored whether ADI treatment significantly affected tube-like (capillary) formation of human umbilical vein endothelial cells. Inhibition occurred in a dose-dependent manner, both in the chorioallantoic membrane and the murine Matrigel plug assay. Inhibition of angiogenesis by ADI was reversed when a surplus of exogenous arginine was provided, indicating that its antiangiogenic effect is primarily due to arginine depletion, although other pathways of interference are not entirely excluded. Arginine deiminase is also shown to be as a potent inhibitor of tumour growth *in vitro* as *in vivo*, being effective at nanogram quantities per millilitre in CHO and HeLa cells. Thus, it could be highly beneficial in cancer therapy because of its two-pronged attack as both an antiproliferative and an antiangiogenic agent.

Arginine deiminase (ADI; EC 3.5.3.6) catalyses the hydrolysis of L-arginine to L-citrulline and ammonia (Miyazaki *et al*, 1990). Enzyme purified from *Mycoplasma arginini* inhibits proliferation of a several types of human cancer cells (Gong *et al*, 2000; Kang *et al*, 2000). Primarily, it would appear through depletion of arginine, because supplementation of the medium with a plentiful supply of arginine generally restores cell proliferation (Komada *et al*, 1997; Kang *et al*, 2000). In addition, ADI displays an antitumour activity *in vivo,* with seemingly minimal side effects (Takaku *et al*, 1992), and is said to inhibit proliferation of human leukaemia cells more potently than asparaginase (Gong *et al*, 2000), which is the only amino-acid-degrading enzyme regularly used in cancer chemotherapy. Nitric oxide (NO) modulates the activity of vascular endothelial growth factor, basic fibroblast growth factor, and matrix metalloproteinases during angiogenesis (Papapetropoulos *et al*, 1997; Sasaki *et al*, 1998), although there are conflicting views (Pipili-Synetos *et al*, 1994; Raychaudhury *et al*, 1996; Phillips *et al*, 2001).

Arginase can also regulate NO level because it is synthesized from L-arginine in a concentration-dependent manner (Gotoh and Mori, 1999). In fact, at least three enzymes destroy arginine. The second enzyme is arginine decarboxylase (Philip *et al*, 2003), and the third is ADI, which we recently showed to have the ability to deplete plasma arginine and suppress LPS-induced NO production, suggesting a potential inhibitory role of ADI in NO-mediated angiogenesis (Noh *et al*, 2002; Thomas *et al*, 2002). Wilm *et al* (1996) purified a protein that strongly inhibited proliferation of vascular endothelial cells, later identified as ADI from *Mycoplasma*. Furthermore, *in vitro* antiangiogenic activity of ADI was recently reported to inhibit migration and microvessel tube formation in human umbilical vein endothelial cell cultures (Beloussow *et al*, 2002). Thus, ADI seems to inhibit tumour growth not only by exhausting the supplies of a vital nutrient (arginine), but also by its antiangiogenic activity via suppression of NO generation. We have therefore examined whether recombinant ADI expressed in *Escherichia coli* inhibits *in vivo* angiogenesis, using several experimental models. We also report on its effects on tumour cell growth in the presence or absence of nitric oxide.

## MATERIALS AND METHODS

### Materials

Cell culture plates were purchased from Corning Costar (Cambridge, MA, USA). Basic fibroblast growth factor (bFGF), endothelial cell growth supplement, heparin, and Drabkin reagent kit 525 were from Sigma (St Louis, MO, USA). M199, isopropyl *β*-D-thiogalactopyranoside (IPTG) and fetal bovine serum (FBS) were from Life Technologies (Grand Island, NY, USA). Matrigel was from BD Biosciences (Bedford, MA, USA), and Sephacryl S-100, DEAE- and arginine-Sepharose resins were from Amersham Pharmacia Biotechnology (Piscataway, NJ, USA).

### Purification of recombinant ADI

The ADI gene was cloned by polymerase chain reaction (PCR) using *M. arginini* genomic DNA. The tryptophan codon TGA in the coding region that corresponds to a stop codon in *E. coli* was changed to TGG by site-directed mutagenesis, using the overlap extension PCR method (Ho *et al*, 1989). The mutated ADI gene was cloned into a pET32a (+) expression vector (designated pET32a-ADI) and transformed into *E. coli* strain BL21. Recombinant ADI overexpressed as inclusion bodies was denatured with 6 M guanidine HCl, refolded in 10 mM potassium phosphate buffer (pH 7.4), and purified by DEAE- and arginine–Sepharose column chromatography (Kang *et al*, 2000).

### Cell culture

*CHO, HeLa, and neonatal foreskin fibroblasts* (9th passage of a primary cell culture) cells were grown as stocks in RPMI 1640 with 10% fetal calf serum and antiobiotics. They were transferred to 24-well plates for growth studies, with the enzyme being incubated in arginine-deficient RPMI 1640 to which 5% dialysed serum and the appropriate test substance was added. Amino-acid additions were made at 400 *μ*M. Negative controls were given no arginine supplementation, but cultures to receive ADI were replete with arginine. The ADI was normalised in its activities such that its IC_50_ was set at a level of 0.1 U ml^−1^, due to changes in specific activity of the enzyme during shipments to Scotland. The specific activities of batches 1 and 2 ADI used were originally 58.3 and 46.4 U mg^−1^ protein, respectively. Effects of cells were seen at 0.1–1 U ml^−1^. Better data were obtained with 2 U ml^−1^, corresponding to higher ID_50_ in the range 0.1–1.0 ng ml^−1^ following normalisation, and agrees well with the data of other groups (e.g. Miyazaki *et al*, 1990; Gong *et al*, 2000). Pegylation of ADI was not carried out for this *in vitro* work. Sodium nitroprusside (Sigma, Poole, UK) was added to the culture medium at a final concentration of between 10 and 250 *μ*M, as appropriate.

*Avoidance of mycoplasmal contamination.* Essential to this study has been the regular checking of cultures for mycoplasma contamination. The most devastating effect on cell cultures can be seen with arginine deiminase released from *M. arginini* as a contaminant. Using the Roche Mycoplasma Detection Kit for four prevalent species of *Mycoplasma* that includes *M. arginini*, cultures were tested before experimentation, according to the manufacturer's instructions. The only cell line that occasionally showed positive *M. arginini* results was HeLa, and fresh uncontaminated cultures were selected for the present work (see Figure 7). However, we also used a supernatant preparation from a pure culture of *M. arginini* provided by Dr Robin Nicholas (Veterinary Laboratories Agency, Weybridge, UK) for comparison with recombinant ADI from *E. coli.*

Growth was measured daily by electronic (Coulter) counting of the cells in three wells per data point. Averages were plotted with one standard deviation of the mean shown in each case. Where bar lines do not appear, they will be within the symbols used on the graphs.

### Tube formation assay

Human umbilical vein endothelial cells (HUVECs) were prepared from freshly delivered cords (Jaffe *et al*, 1973) and confirmed by immunocytochemical staining for Factor VIII. The cells were cultured in 25 cm^2^ flasks in M199 media supplemented with 10% FBS, 100 U ml^−1^ penicillin, 100 *μ*g ml^−1^ streptomycin, 50 *μ*g ml^−1^ endothelial cell growth supplement, and 50 *μ*g ml^−1^ heparin in a 37°C incubator with humidified atmosphere containing 5% CO_2_. The cells between passages 3 and 5 were used for *in vitro* experiments. Capillary-like tube formation assay was performed as described previously (Grant *et al*, 1992), but with the following modifications. The 48-well plates were coated with 200 *μ*l Matrigel (10 mg ml^−1^) by incubating at 37°C for 1 h. Human umbilical vein endothelial cells were suspended in M199 media supplemented with 10% FBS, and plated onto a layer of Matrigel at a density of 4 × 10^4^ cells well^−1^ with or without ADI. The plates were incubated for a further 18 h at 37°C, and capillary-like tube formation was observed microscopically, their area of being measured using Image-Pro Plus software (Media Cybernetics, Silver Spring, MD, USA).

### CAM assay

Fertilised chicken eggs were incubated at 37^o^C in a humidified incubator. On the third day of incubation, 2 ml of albumin was aspirated from the eggs with an 18-gauge hypodermic needle to detach the developing CAM from the shell. On the fourth day, the shell was punched out to make a window and sealed with a cellophane tape. Thermanox coverslips (Nunc, Naperville, IL, USA) coated with or without 0.046 U ADI were applied to the CAM surface through the windows for 2 days. After injection of an appropriate volume of 10% fat emulsion (intralipose) into the chorioallantois, angiogenesis in CAM was observed under a microscope, with >20 eggs being used for each assay.

### *In vivo* Matrigel plug assay

Male C57BL/6 mice (7 weeks old) were supplied from Daehan Biolink Co. (Seoul, South Korea). Matrigel plug assay in mice was performed as described previously (Passaniti *et al*, 1992). Briefly, 0.4 ml of Matrigel containing 0.46 U ml^−1^ ADI, 50 ng ml^−1^ bFGF, and 60 U ml^−1^ heparin were injected subcutaneously near the abdominal midline of the mice using a 25-gauge needle. Control animals were subjected to subcutaneous injection of Matrigel mixture without ADI. Where heparin and bFGF were used, these were added along with the Matrigen inoculation. After injection, the Matrigel rapidly formed a solid gel at the site of injection. Plugs were separated from the abdominal wall after 5 days and fixed with 3.7% formaldehyde in phosphate-buffered saline. The formalin-fixed Matrigel samples were embedded in paraffin and examined with Masson-trichrome stain. To evaluate new blood vessel formation, haemoglobin content was measured with a Drabkin reagent kit 525. The concentration of haemoglobin was calculated from a known amount of haemoglobin assayed in parallel. The experiment was repeated twice with five mice in each group.

## RESULTS

### Expression and purification of recombinant ADI

**Figure 1:**
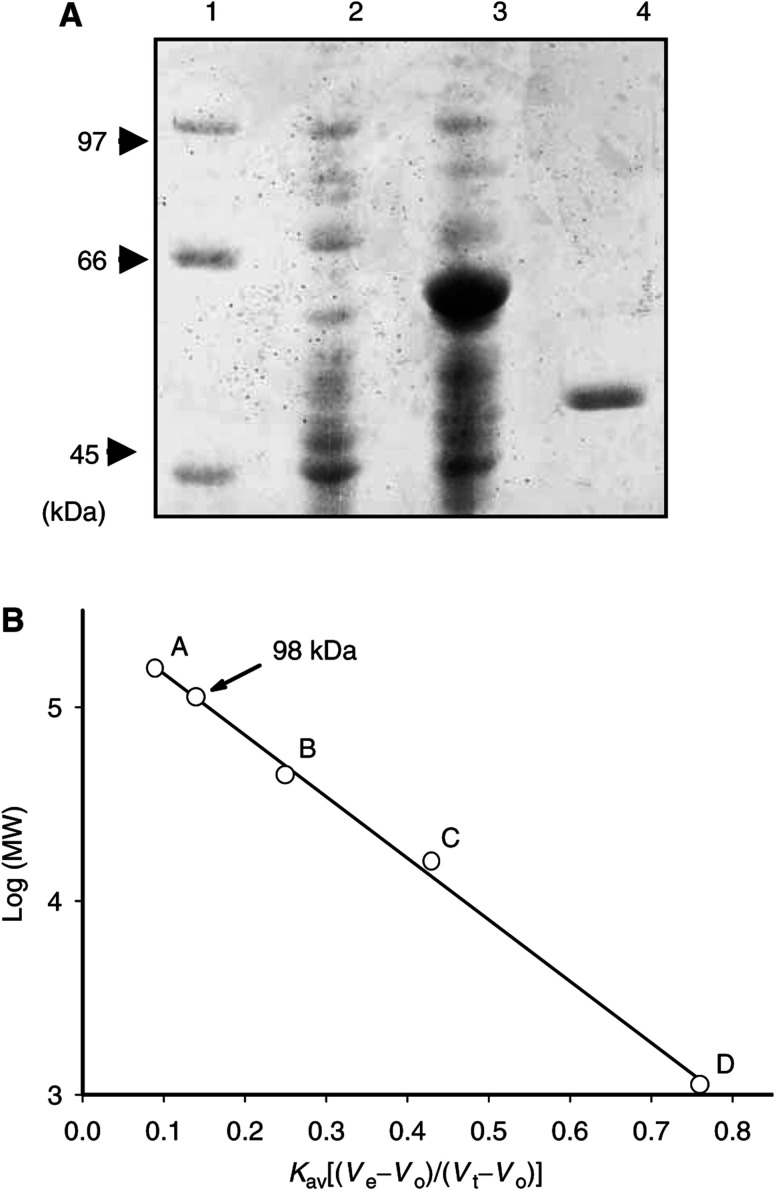
SDS–PAGE and gel filtration analysis of recombinant ADI. (**A**) Thioredoxin–ADI fusion protein expressed in *E. coli* was resolved by 10% SDS–PAGE. Lane 1; molecular weight marker proteins, lane 2; crude extract without IPTG induction, lane 3; inclusion body after IPTG induction, lane 4; elute from arginine-affinity column chromatography. (**B**) Molecular weight of the self-digested ADI was determined by calibrated Sephacryl S-100 gel filtration; standard markers A: bovine gamma globulin (*M*_r_ 158 kDa), B: chick ovalbumin (*M*_r_ 44 kDa), and C: equine myoglobin (*M*_r_ 17 kDa), D: vitamin B_12_ (*M*_r_ 1.35 kDa).

Recombinant ADI gene was expressed in *E. coli* as a thioredoxin fusion protein with His-tag and S-tag, using a pET32a (+) expression vector. Thioredoxin–ADI fusion protein was accumulated in the cytoplasm as inclusion bodies and its molecular weight was estimated at 64 kDa (Figure 1A;

this is included here to show the authenticity of the batches of enzyme prepared for the present work). However, the fusion protein tended to be self-degrading during the process of denaturation and refolding for purification. Although the precise cleavage site of self-digestion in the linker region was not determined, enough enzyme activity was retained to hydrolyse arginine. However, this gave a much lower specific activity than for recombinant arginase (Philip *et al*, 2003). Our recombinant ADI was estimated at 49 kDa on SDS–PAGE and 98 kDa by Sephacryl S-100 gel filtration (Figure 1A and B), indicating the same dimeric structure as native ADI purified from *M. arginini* (Takaku *et al*, 1992). After DEAE–Sepharose and arginine-affinity column chromatography, ∼5 mg active ADI was purified from 1 l of culture.

### Inhibition of capillary-like tube formation of HUVECs by ADI

**Figure 2:**
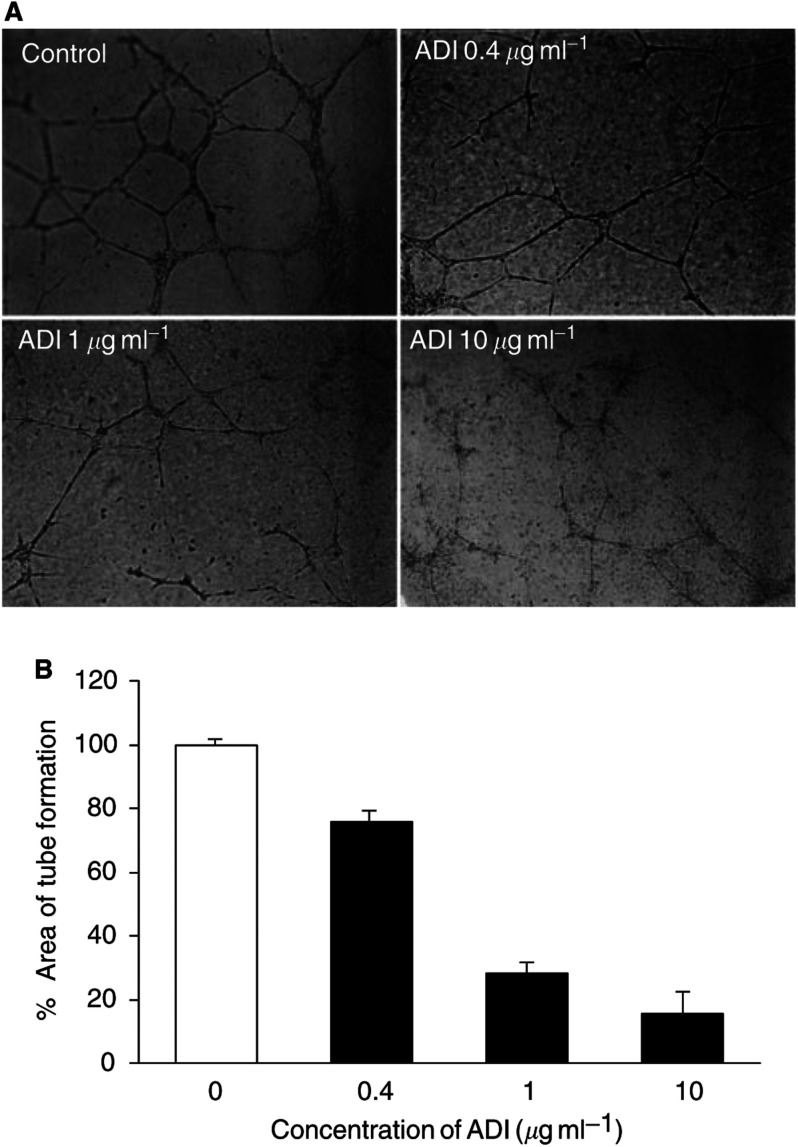
Inhibitory effect on tube formation of human endothelial cells by ADI. HUVECs were plated on Matrigel-coated wells at a density of 4 x 10^4^ cells well^−1^ with various amounts of ADI (0.012–0.46 U ml^−1^; i.e. 0.4–10 *μ*g ml^−1^). The plates were further incubated for 18 h at 37°C, and capillary-like tube formation was photographed using microscope (**A**). The area showing capillary-like tube formation was measured by Image-Pro Plus software (Media Cybernetics, Silver Spring, MD, USA) and represented as percentage of the control value (**B**). The values are means±s.e. (*n*=3 independent experiments performed in duplicate).

**Figure 3:**
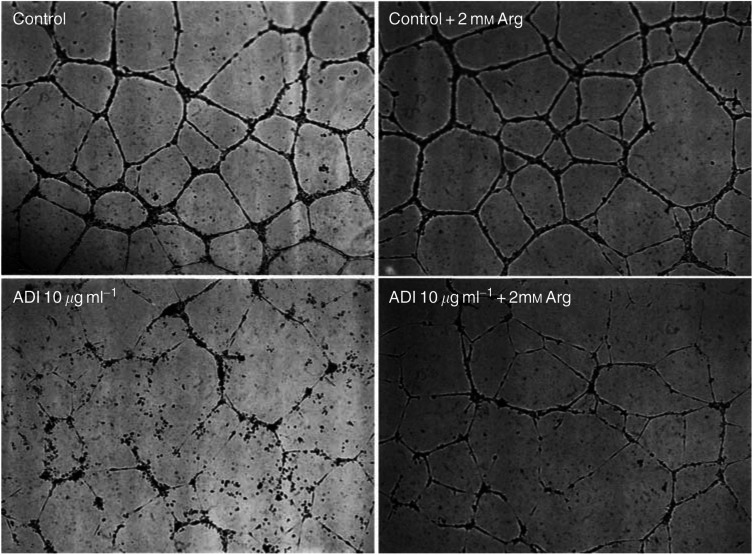
Recovery of angiogenesis by supplement of arginine. Arginine (2 mM) was added to the culture of HUVECs treated with 0.46 U ml^−1^ (10 *μ*g ml^−1^) ADI to examine the antiangiogenic effect by arginine depletion. Tube formation suppressed by ADI treatment was restored after supplement of arginine. Each sample was assayed in triplicate and the assays were repeated twice.

To examine the effect of ADI on tube formation by vascular endothelial cells, HUVECs were plated on the Matrigel-coated culture dishes and treated with ADI from 0.012 to 0.46 U ml^−1^. Capillary-like tube formation, an indication of *in vitro* angiogenesis, was well developed in control HUVECs after 18 h of incubation (Figure 2A

). Arginine deiminase treatment significantly inhibited the capillary-like tube formation of HUVECs in a dose-dependent manner compared with controls (Figure 2A). The percentage of tubule area after 18 h treatment from 0.012 to 0.46 U ml^−1^ ADI ranged from 76 to 16% of the control values (Figure 2B). To characterise the antiangiogenic effect of ADI, 2 mM arginine was added to the culture media containing 0.46 U ml^−1^ ADI. Tube formation resumed following the exogenous arginine, whereas no new tube formation appeared in controls not supplemented with arginine (Figure 3

).

### Inhibition of angiogenesis by ADI *in vivo*

**Figure 4:**
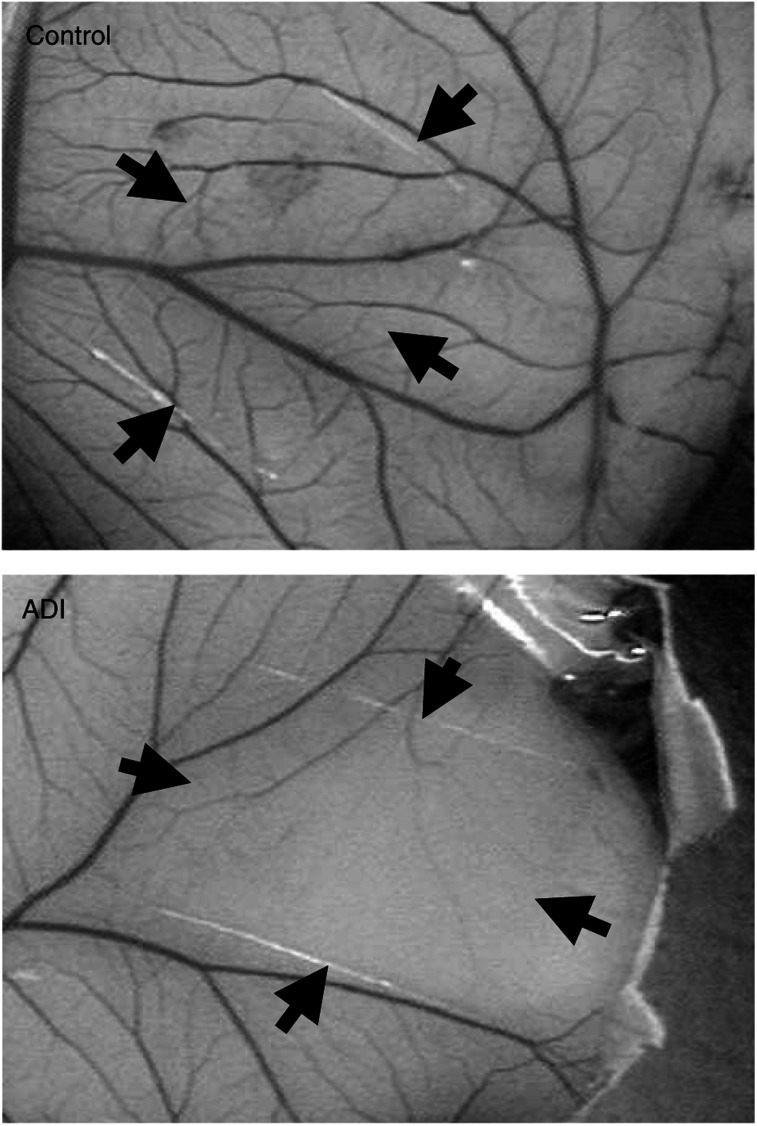
Antiangiogenic effect of ADI on the chick CAM. Arginine deiminase (0.046 U) was loaded in Thermanox coverslips, air-dried and applied to the CAM surface of fertilised eggs (Materials and methods). An avascular zone of the CAM beneath the disk (surrounded by arrows) was produced 2 days after treatment of ADI, but not in control disk. At least 20 eggs were used for each assay.

**Figure 5:**
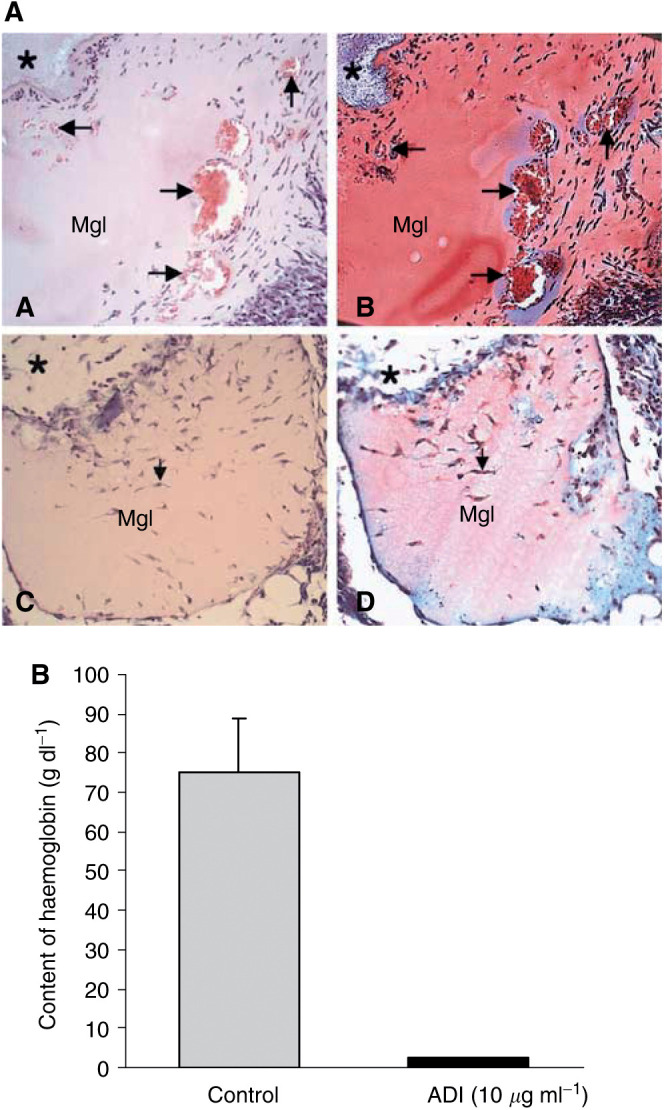
Inhibition of angiogenesis by ADI *in vivo* mouse Matrigel assay. Matrigel (0.4 ml) containing 50 ng ml^−1^ of bFGF and 60 U ml^−1^ of heparin in combination with or without 0.46 U ml^−1^ ADI was subcutaneously injected near the abdominal midline of the mice. (**A**) Histological analysis of Matrigel implants (for experimental procedures, see Materials and methods). Matrigel without ADI (A and B) showed formation of blood vessels with various sizes (arrows) forming in the Matrigel. Inside the vessel, red blood cells were observed (red colour in the vessel). However, ADI treatment clearly inhibited blood vessel formation (C and D). ^*^Indicates connective tissues surrounding Matrigel implants. (A and C: haematoxylin–eosin staining, B and D: Masson-Trichrome staining). Original magnification x100. (**B**) Haemoglobin content in the Matrigel was measured with Drabkin reagent kit 525 to evaluate blood within the vessels formed 5 days after injection, calibrated against a known amount of haemoglobin in parallel. ADI potently inhibited growth factor-induced angiogenesis by 97%. Each value represents the mean±s.e.of five ADI-treated animals and seven per control group.

To determine the *in vivo* antiangiogenic effect of ADI, CAM and Matrigel plug assays were carried out. At 0.046 U ml^−1^, ADI significantly inhibited chick embryonic angiogenesis, producing an avascular zone beneath the disk in 88% of the ADI-treated group, whereas control CAMs treated with saline showed no disturbance of angiogenesis (Figure 4

). In addition, ADI inhibition was quantitatively estimated using the *in vivo* mouse Matrigel plug assay. After 5 days of implantation, the control – Matrigel plugs containing bFGF and heparin without ADI appeared dark red in colour due to massive formation of blood vessels. Matrigel plugs treated with 0.46 U ml^−1^ ADI in combination with bFGF and heparin remained pale, indicating no blood vessel formation (Figure 5A

). Haemoglobin content in the Matrigel plugs was taken as a measure of vasculature formation; the content in control Matrigel without ADI was 75.2±13.8 g dl^−1^ (*n*=5), whereas Matrigel with 0.46 U ml^−1^ ADI was 2.5±0.1 g dl^−1^ (*n*=7, Figure 5B), indicating that ADI inhibited growth factor-induced angiogenesis in this model by ∼97%.

In experiments testing the effect of the spontaneous nitric oxide generator, sodium nitroprusside, both HUVEC and fibroblasts in angiogenesis co-culture (Bishop *et al*, 2000) tolerated the upper range of our treatment very well (10–500 *μ*M), and there was no obvious effect of tubule formation throughout their period of development (14 days) in the co-culture system, whether or not sodium nitroprusside was added to the medium being replaced every other day (data not shown).

### Inhibition of tumour cell growth by ADI

**Figure 6:**
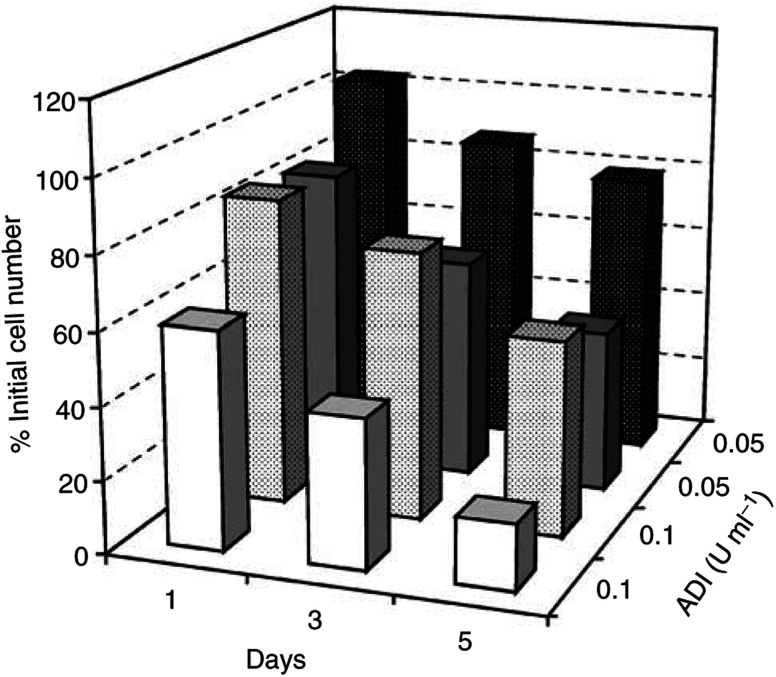
Comparison of the effect of ADI on CHO and HeLa cell growth. CHO and HeLa cells were treated with comparable levels of ADI (0.05 and 0.1 U ml^−1^) and the data giving cell number relative to the start (50 000 cells per well) are recorded as means of triplicate sample after 1, 3, and 5 days. In both cases, the CHO cells were more vulnerable than HeLa cells (HeLa cultures have stippled bars, CHO are plain).

**Figure 7:**
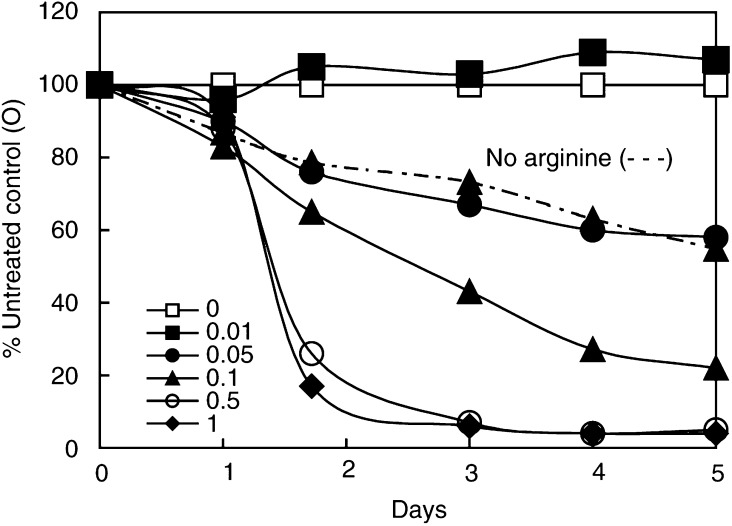
Dose–response curves for ADI on CHO cells with (solid lines) and without arginine (broken lines). Effect of increasing concentrations of ADI from 0.01 to 1 U ml^−1^ (see inset box) on CHO cells from an inoculum of 50 000 cells per well (100%), and expressed daily as a percentage relative to the control (□). The dashed curve (- - ▴ - -) gives data for a culture in which 0.1 U ml^−1^ ADI were added but *without* 400 *μ*M arginine being present. (Note that this curve is much less steep and some of the cells persist and are viable at the end of the experiment (5 days), whereas those given arginine and enzyme were dead within 3 days.)

We reasoned that if inhibition of tumour cell growth by ADI *in vitro* was primarily and largely due to depletion of arginine, the responses of two of the three carefully chosen cell lines would be decidedly different (Wheatley and Campbell, 2003). We have tested ADI on many cells lines and found that it inhibits growth and/or kills cells as effectively as arginase treatment (Scott *et al*, 2000), but we have confined our experiments here to these two because they represent the ends of a spectrum of the ability of cells to utilise citrulline. Thus, ADI should be highly effective against cells that are incapable of metabolising citrulline *in lieu* of arginine, the selection therefore being the CHO cell line. However, whether citrulline is produced by ADI or exogenous citrulline is added to the culture medium of cells that can metabolise it to arginine, ADI should be less effective. HeLa cells can metabolise exogenous citrulline, although a concentration ∼2 times greater than arginine is needed to sustain a comparable growth rate (Philip *et al*, 2003). Data from a comparative experiment with ADI clearly show its greater damaging action on CHO than HeLa cells (Figure 6

). The sensitivity of CHO to the ADI in Figure 7

includes curves for cultures set up at time zero in arginine-free medium, to which arginine was either added at the same time as the enzyme at two comparable ADI levels (dashed lines) or omitted (solid lines). In cultures that initially had arginine-replete medium, ADI was more toxic than those without (see Discussion).

**Figure 8:**
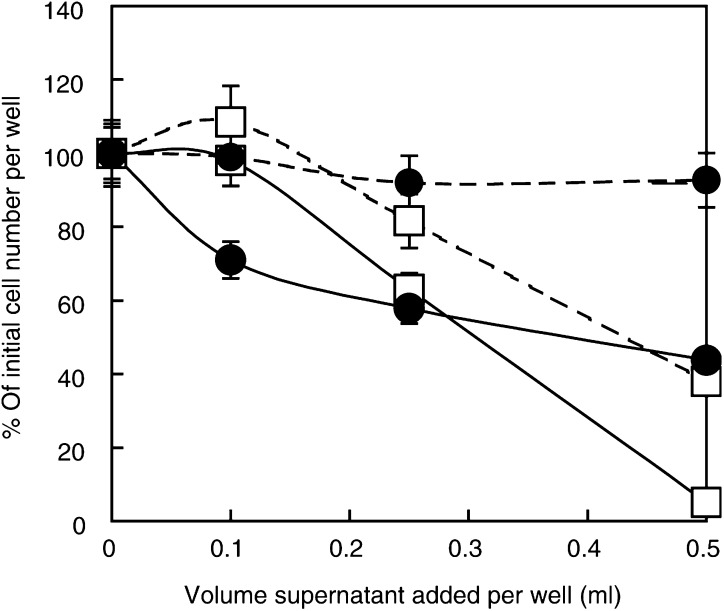
Action of ADI on fetal foreskin fibroblasts and HeLa cells. HeLa and FF9 cells were cultured in 24-well plates from a 25 000 cells per well inoculum. After 1 day, they were treated with supernatant from a *M. arginini* culture prepared for us by Dr Robin Nicholas. Argenine deiminase activity was 0.2–0.25 U ml^−1^ in the media added to the cultures. The cultures were left 3 days before the number of cells were estimated and expressed as a percentage of the controls taken as 100% of the initial value. The square symbols refer to HeLa cells, with the solid line being cultures with arginine present, while the negative controls (no arginine, but the same amount of ADI) has the dashed line. The symbol for FF9 fibroblasts is a closed circle, and the dashed and the solid lines are as for the HeLa cultures. In both cases, cells did worse where arginine was degraded by ADI than when it was absent. Means are plotted of triplicate values and the bar lines give 1 s.d.

The data accord well with observations on cell lines made where contamination with *M. arginini* has occurred, as described below. We checked the effect of supernatants from cultures of *M. arginini* on HeLa cells and fetal fibroblasts to show that medium with known high levels of ADI were inhibitory not just to HeLa cells, but to normal cells (Figure 8

). This experiment confirms that the enzyme expressed was ADI, and that when this organism invades HeLa cultures, the cells fail to thrive after normal subculturing procedures and quickly die. We therefore took an authentic preparation of ADI as the supernatant of a pure-type cultures collection of *M. arginini* from Dr Robin Nicholas (National Microbiological Standards Centre, Weybridge, UK), and show here that normal cells that convert citrulline to arginine (see Philip *et al*, 2003) and grow more slowly than many tumour cells lines are nevertheless inhibited by ADI at approximately comparable doses, although cell death is much less extensive in the normal cells than in HeLa cells. This contrasts with fibroblasts deprived of arginine by medium formulation, in which virtually no cell death occurred over 3 days of incubation (Figure 8; see Discussion).

We also used HeLa cells to show that, where arginine is depleted and a restricting level of citrulline slows or arrests growth due to ADI, this effect occurs independently of NO reduction. This was done by carrying out studies on HeLa cells in the presence of ADI at lower levels of arginine (100–250 *μ*M) with or without the presence of a NO donor, sodium nitroprusside, present at equivalent concentrations, which made no difference to the resulting depression of growth (therefore data not shown; see Discussion). N.B. At 100–250 *μ*M, a few cells were damaged in the early treatment of controls, but there was no increased (synergistic or additive) effect of ADI treatment.

## DISCUSSION

### Antiangiogenic effects

Recombinant ADI from *E. coli* inhibits angiogenesis in two different experimental systems. Arginine deiminase suppressed *in vitro* capillary-like tube formation of vascular endothelial cells and *in vivo* neovascularisation in the CAM and the Matrigel plug assays in the mouse. This microbial enzyme, ADI, specifically hydrolyses arginine to citrulline, leading to depletion of an essential amino acid that is utilised not only for protein synthesis, but also is a substrate for NO, polyamine, proline, and glutamate production in mammals (Wu and Morris, 1998). Deprivation of extracellular arginine by ADI treatment inevitably disrupts a number of biochemical pathways, including some intracellular regulatory signals for endothelial cell growth. Although an antiangiogenic activity of NO has been reported (Raychaudhury *et al*, 1996; Philip *et al*, 2001), several investigators showed a positive association of NO level with angiogenic activity (Papapetropoulos *et al*, 1997; Murohara *et al*, 1998). Interestingly, overexpression of arginase, another arginine-degrading enzyme, markedly inhibits NO synthesis and enhances polyamine production in endothelial cells by modulating intracellular arginine availability (Li *et al*, 2001). Thus, it can be assumed that the antiangiogenic effects of ADI are associated with NO suppression resulting from depletion of arginine. Inhibition of capillary-like tube formation of vascular endothelial cells by ADI treatment is more likely to be due to the depletion of arginine, since exogenous arginine supplementation reversed its inhibitory effects. We found no evidence that the lack of NO production as a consequence of arginine deprivation has any significant effect of the angiogenic process in the presence or absence of ADI.

In addition to NO suppression *per se* being not relevant in this context, arginine depletion by ADI treatment would alter levels of polyamines, proline, and glutamate. As polyamines are essential for proliferation of endothelial cells (Morrison *et al*, 1995), their decreased synthesis may also affect angiogenesis. In fact, *α*-difluoromethylornithine (DFMO), an irreversible inhibitor of ornithine decarboxylase, a rate-limiting enzyme in the polyamine biosynthesis, inhibited proliferation of HUVECs (Takahashi *et al*, 2000). Thus, decreased polyamine synthesis due to ADI treatment will no doubt exacerbate the antiangiogenic action.

### Antitumour action

With regard to the suppression of tumour cell growth by ADI, our findings clearly indicate that cells behave the same way whether or not NO is present, unlike endothelial cells. The inhibitory action of the enzyme will be principally due to a drastic limitation of arginine that accounts for CHO and HeLa cells being arrested in growth, and both of these lines undergo considerable death because malignant cells fail to reach quiescence (Scott *et al*, 2000). However, this does *not* necessarily explain the *damaging* as opposed to arresting action of ADI on normal fibroblasts (Figure 8). Furthermore, we see the damaging action of ADI being manifest here not so much *in the absence of arginine* (in the negative controls), as when it has acted upon arginine from the start of the incubation. Although we have not proved that the release of *nascent* NH_3_ (not ammonium ions) as the by-product of arginine degradation by ADI is responsible, this interpretation seems reasonable. When we consider the effect on CHO and HeLa cells (Figure 6-8), it is noteworthy that tumour cells are particularly affected at critical ADI concentrations, once again presumably through the release of nascent NH_3_. When arginine is present rather than absent and ADI is added critically at ∼0–2 to 0.4 U ml^−1^, both elements are clearly involved, and thus it is not an ‘either/or’ situation, but one where both apply, resulting in the more devastating effect of ADI in the *presence* of arginine. This is in good agreement with the *data* of Gong *et al* (2000) and others reporting massive cell death with ADI (Komada *et al*, 1997), although not entirely with their *interpretation*.

### Native ADI

We also checked that the purified enzyme prepared from *E. coli* possessed all the characteristics of authentic ADI originating from a pure strain of *M. arginini* (Figure 8). The evidence is a salutary reminder that cultures of cells need to be regularly checked for *M. arginini* because the ADI released from these organisms in considerable quantities devastates cultures of both normal and malignant phenotypes.

It is most unlikely that NO being produced through the action of other enzymes on arginine (NO synthase) plays a role in tumour cell death. Sodium nitroprusside at concentrations that should release very considerably greater amounts of NO into a closed culture system would not be expected to show toxicity until ∼100 *μ*M, and it is already known that some cell types such as rat lung epithelial cells (Bundschuh *et al*, 1995) tolerate 2.6 mM sodium nitroprusside. We found some occasional cell damage soon after treatment at 100 *μ*M and a little more at 250 *μ*M, but this was neither additive nor synergistic in nature. The absence of NO would not in any way have been responsible for helping cells to survive the effect of arginine deprivation induced by ADI.

Previously, ADI was reported to have *in vivo* antitumour activity (Takaku *et al*, 1992), especially in leukaemia. Gong *et al* (2000) claimed it was more effective than L-asparaginase, but did not to take into account the widely disparate specific activities of the two enzymes (Wheatley and Campbell, 2002), comparing them only on a mass per volume basis. Arginine is one of the essential nutrients for mammalian cells and the first amino acid depleted by cell metabolism in culture (Wheatley *et al*, 2000). The rapid depletion of arginine may be partly due to the fact that arginine is less efficiently recycled (especially residues recovered from catabolised proteins) by cultured cells than other amino acids, but the kinetics of this recycling have yet to be determined in animals where tumours have been inhibited by ADI. The reutilisation of citrulline following ADI action remains a worrying aspect of its efficacy, but will always depend on an intact urea cycle (lacking in many tumour cell types, Wheatley and Campbell, 2003).

### General conclusions

Upon deprivation of arginine, normal diploid fibroblasts having stringent check points of cell cycle can survive for several days in a quiescent state (G0) after exiting from cell cycle, whereas tumour cells lacking stringent regulatory cell cycle checkpoint fail to enter quiescence and continue to divide until cell death intervenes through some form of imbalance and/or apoptosis (Scott *et al*, 2000; Wheatley *et al*, 2000). Thus, deprivation of arginine by ADI could induce detrimental pleiotypic responses and lead to prompt tumour cell death by disrupting the biochemical pathways of the cell cycle. In addition to its antiproliferative effect on tumour growth, we demonstrated that ADI suppressed angiogenesis by inhibiting both tube formation of endothelial cells and neovascularisation in CAM and Matrigel plug assay. Since the latter does not require cell proliferation, the action of arginine degrading enzyme may play other roles than simply reducing the availability of arginine as a precursor in these and other pathways that have not already been mentioned. Solid tumours could not proliferate continuously without recruiting a blood supply by angiogenesis (Folkman *et al*, 1995). These results suggest that ADI could synergistically inhibit *in vivo* solid tumour growth by the combined action of antiproliferative activity through the depletion of arginine as a nutrient and its own antiangiogenic activity. However, it is also clear that the different strands of this complex process need to be teased out and subjected to a more thorough analysis, especially the rate of citrulline recycling.
